# Using ^77^Se-Labelled Foliar Fertilisers to Determine How Se Transfers Within Wheat Over Time

**DOI:** 10.3389/fnut.2021.732409

**Published:** 2021-10-15

**Authors:** Chandnee Ramkissoon, Fien Degryse, Scott Young, Elizabeth H. Bailey, Michael J. McLaughlin

**Affiliations:** ^1^Fertiliser Technology Research Centre, School of Agriculture, Food and Wine, University of Adelaide, Glen Osmond, SA, Australia; ^2^School of Biosciences, University of Nottingham, Loughborough, United Kingdom

**Keywords:** selenium, wheat, speciation, biofortification, foliar fertilisation

## Abstract

Foliar selenium (Se) fertilisation has been shown to be more efficient than soil-applied fertilisation, but the dynamics of absorption and translocation have not yet been explored. An experiment was undertaken to investigate time-dependent changes in the absorption, transformation, and distribution of Se in wheat when ^77^Se-enriched sodium selenate (Se_fert_) was applied to the leaves at a rate of 3.33 μg Se per kg soil (equivalent to 10 g ha^−1^) and two growth stages, namely stem elongation, Zadoks stage 31/32 (GS1), and heading stage, Zadoks stage 57 (GS2). The effect of urea inclusion in foliar Se fertilisers on the penetration rates of Se was also investigated. Wheat was harvested at 3, 10, and 17 days and 3, 10, and 34 days after Se applications at GS1 and GS2, respectively. Applying foliar Se, irrespective of the formulation, brought grain Se concentration to a level high enough to be considered adequate for biofortification. Inclusion of N in the foliar Se solution applied at an early growth stage increased recoveries in the plants, likely due to improved absorption of applied Se through the young leaves. At a later growth stage, the inclusion of N in foliar Se solutions was also beneficial as it improved the assimilation of applied inorganic Se into bioavailable selenomethionine, which was then rapidly translocated to the grain. The practical knowledge gained about the optimisation of Se fertiliser formulation, method, and timing of application will be of importance in refining biofortification programs across different climatic regimes.

## Introduction

Micronutrient deficiencies affect one in three people globally (1) as a result of intake patterns or absorption rates that fall below the level required to sustain good health and development (2). Selenium (Se) is one such micronutrient that is currently consumed at lower-than-recommended levels in many parts of the world. Combs (3) estimated that 0.5–1 billion people worldwide were at risk of Se deficiency diseases as a result of inadequate dietary Se intake.

Selenium is an essential nutrient for both humans and animals (4). It has been shown to have antiviral effects, be beneficial for reproduction, and lower autoimmune thyroid disease risks. More recently, its role as an antioxidant and a potential anticarcinogen has been appraised (5, 6). Although inadequate Se intake can cause general poor health, extremely low levels of Se can cause specific deficiency diseases such as Keshan (cardiomyopathy) and Kashin–Beck (an osteoarthritis disorder); these are seen, for example, in some regions of China and Siberia (7, 8). However, Se can also be toxic if ingested at higher-than-recommended levels. An excess of Se in the body, resulting in “selenosis,” is characterised by the loss of hair and nails, and general fatigue (9). The current daily recommended intake of Se is set at 55 and 70 μg person^−1^ for women and men, respectively; more generally, a dietary Se intake range of 40–400 μg day^−1^ is considered safe (10). As a result of increasing concern about the inadequacy of Se intake in many locations around the world, research has, in recent decades, focused on ways to improve dietary Se levels sustainably to preempt or alleviate Se deficiency.

Agronomic biofortification is a term describing the process through which the concentration of micronutrients in edible parts of staple crops is increased through the application of fertilisers enriched with trace elements (2). The efficacy of Se fertilisers to fortify crops depends on several factors, including the chemical form of Se used its application rate, and its method. Selenium is most commonly applied in its oxidised inorganic forms, such as selenate (Se^VI^) or selenite (Se^IV^) either to the soil or to the canopy (foliar fertilisation) or as a combination of soil and foliar (11). When soil-applied, selenate is often the preferred source for biofortification because of its higher mobility in the soil and plants. Selenate is highly mobile in the xylem and accumulates in the edible parts of plants before being converted to bioavailable organic forms such as selenomethionine (SeMet). By contrast, Se^IV^, despite rapid uptake into roots, is generally converted more rapidly to organic forms and accumulates in roots (12). The efficacy of soil-applied Se fertilisers is largely dependent on the chemical speciation of Se and the physicochemical properties of the soil (13). Soil components such as metal oxides, clays, and soil organic matter (SOM) have the potential to adsorb Se strongly, especially Se^IV^, resulting in reduced mobility and availability of Se in the soil. Selenate, on the other hand, adsorbs *via* a weaker mechanism and hence is more mobile and bioavailable than Se^IV^. However, selenate is also more prone to leaching than Se^IV^, especially in acidic environments (14). Moreover, the biogeochemical behaviour of Se in soils is influenced by the presence of environmental microorganisms in the soil, particularly arbuscular mycorrhizal fungus (AMF), such that, it is essential to consider plant–bacteria-fertiliser interactions in soils to optimise Se biofortification (15).

In contrast to soil-applied fertilisers, foliar fertilisers tend to be more efficient due to the reduced losses to the environment by leaching and/or adsorption to soil particles (16, 17). In contrast to soil application, foliar-applied nutrients are absorbed through the leaf epidermis and transferred to the rest of the plant *via* the phloem (18, 19). Effectively, Ros et al. (16) showed that foliar fertilisation could be on average eight times more efficient than Se application to soil. For example, they found that an application rate of 30–60 g ha^−1^ Se^VI^ to the soil would be needed to increase grain Se concentration from 0.07 to 0.1 mg kg^−1^ compared with just 4.5–10 g ha^−1^ Se^VI^ when the foliar application of Se was used (16). However, foliar fertilisers may also be prone to losses, for example, through leaf runoff following rainfall. More information about the penetration rates of foliar-applied Se fertilisers into plants, and subsequently transfer to edible parts of the plant may be useful to mitigate such losses and optimise foliar Se fertilisation.

In previous studies, we demonstrated that the concentration of bioavailable Se (selenomethionine) in wheat grain subject to foliar Se applications could be increased through the addition of small amounts of nitrogen (N), for example, urea (20). Although the exact mechanism for this improved efficiency is not yet fully understood, it was suggested that N aided Se assimilation into organic Se forms in the leaves, which were then transported to the grain. There is also limited literature about the optimum timing of foliar Se application for biofortification. Lyons (17) suggested that the application of nutrients such as Se and I are best made between the booting and early milk stages, preferably around the heading stage, to maximise the area of canopy available for fertiliser interception and uptake. Understanding how Se transfers from the point of application to the rest of the crop at different growth stages may be useful in planning Se fertilisation tactics to optimise crop uptake.

In this study, we aimed to determine the time-dependent changes in Se absorption, assimilation, and transfer to the aboveground biomass, following the application of ^77^Se-labelled selenate fertilisers to wheat leaves. The use of stable isotope Se tracers, such as enriched ^77^Se, enables the simultaneous determination of native (soil-derived) and applied Se sources in both plant and soil systems (21, 22). The partitioning of the applied ^77^Se-fertiliser in wheat was assessed when different (a) foliar treatments (Se ± N) and (b) application timings (growth stages) were employed. This study provided practical information about the uptake and transformation of foliar-applied Se in wheat, which farmers could use to manage fertiliser application methods and timing to optimise Se biofortification.

## Materials and Methods

### Soil

Sandy loam topsoil was used for the pot trial (Table 1). The soil was air-dried and sieved to <2 mm prior to characterisation. Soil pH and electrical conductivity (EC) were measured in a 1:2.5 soil-to-solution suspension on an automated Skalar pH/EC system. Soil organic matter content was estimated by the loss-on-ignition method (23). Particle size analysis was determined by laser granulometry following treatment with 40% hydrogen peroxide (H_2_O_2_), as described in Mathers et al. (22). Extractable P and S (mg kg^−1^) were determined by the method developed by Olsen et al. (24) and Blair and Lefroy (25). The water holding capacity (WHC) of the soil was determined using ceramic tension plates and hanging water columns (26).

**Table 1:** Physicochemical properties of the soil used in the experiment.

pH (water)	7.9
Electrical conductivity (μS cm^−1^)	1,300
Organic matter (%)	4.1
Clay (%)	13
Sand (%)	72
Extractable P (mg kg^−1^)	3.0
Extractable S (mg kg^−1^)	18

### Pot Trial

The pot trial was set up in spring (April–May 2019) in a glasshouse at the University of Nottingham Sutton Bonington Campus (United Kingdom). The crops were grown under natural light conditions, which averaged ~6 h daily. Five seeds of spring wheat (*Triticum aestivum* cv. Willow) were sown directly into free-draining pots containing 1.8 kg soil and thinned to two plants per pot 3 weeks later. Plants were fertilised with 5 ml of an ammonium nitrate solution (16.4 g L^−1^ NH_4_NO_3_) at stem extension and head emergence. No additional basal fertilisation was applied to the soil as sufficient plant-available nutrients were present (Table 2). Pots were arranged in a randomised block design and watered to an estimated weight of 60% WHC of the soil using Milli Q water (18.2 MΩ cm) throughout the experiment. All treatments were replicated four times.

**Table 2:** The dry matter yield of aboveground plants harvested 3, 10, and 17 days after Se application at stem elongation (GS1) and 3, 10, and 34 days after Se application at the heading stage (GS2) (SE in brackets; *n* = 4).

**Growth stage (GS)**	**Days after sowing (DAS)**	**Harvest time following Se_**fert**_ application**	**Dry matter yield^**†**^**
	***D***	***d***	**g pot^**−1**^**
1	66	3	3.16 (0.08)^e^
	73	10	4.65 (0.20)^d^
	80	17	5.92 (0.29)^c^
2	122	3	18.9 (0.56)^b^
	129	10	21.7 (0.50)^a^
	153	34	22.2 (0.44)^a^

a−e *Indicate significant differences (p <0.05)*.

### Foliar Selenium Fertiliser Application

Selenium fertilisers (Se_fert_) were prepared from a ^77^Se-enriched sodium selenate solution (259 mg L^−1^ ^77^Se^VI^). Selenium was applied at a single, realistic rate of 3.33 μg kg^−1^; this is equivalent to ~10 g ha^−1^, based on a 20-cm depth of topsoil and 1.5 g cm^−3^ bulk density. Three fertilser treatments were used: (i) foliar-applied Se only (F.Se); (ii) foliar-applied Se with a 2% w/v N source in the form of urea (Sigma–Aldrich, 99–100% purity, United Kingdom) (F.Se+N); (iii) control (Ctrl) where neither Se nor N was applied. The foliar Se+N solution was prepared by dissolving 0.21 g of urea in a solution with a ^77^Se concentration of 180 mg L^−1^. The foliar solutions contained 0.5% surfactant (Triton-X 100; Sigma–Aldrich), which served to reduce the surface tension between the droplets and the leaf, thereby promoting fertiliser absorption. Foliar solutions were applied as four drops of 5 μl volume droplets to the youngest flag leaves of each plant (two plants per pot). For the control treatment, water with 0.5 % surfactant was applied in a manner similar to foliar Se solutions.

The application was either at growth stage 1 (GS1), which was at stem elongation [growth stage 31/32 on the Zadoks scale and 63 days after sowing (DAS)] or GS2, which was at head emergence [Zadoks stage 57 and 119 DAS; (27)].

The surface of the soil was covered with cling film for a week following foliar fertiliser application and care was taken not to irrigate the plants immediately after foliar fertilisation to prevent any potential runoff into the soil.

### Plant Harvest

The aboveground biomass of the wheat plants was harvested at 3, 10, and 17 d (H3, H10, and H17) after fertiliser application at GS1 and 3, 10, and 34 days (H3, H10, and H34) after fertiliser application at GS2. For the plants treated at GS2, wheat heads were harvested separately from the straw and, for the last sampling (H34), wheat heads were further hand-threshed to separate the wheat grains. All the foliar-treated leaves were harvested separately from the straw, washed in 0.1% v/v detergent, and then rinsed with Milli Q water (28). Water rinses were saved to analyse for any unabsorbed applied Se_fert_. After harvest, all plant parts were dried at 50°C for 72 h or until the constant dry weight was achieved. The dry weights of the different plant parts were recorded. Subsequently, plant material was ground using a centrifugal mill (model ZM 200, Retsch, Germany) fitted with a 0.5 mm titanium screen and stored under ambient conditions prior to digestion and chemical analyses.

### Selenium Analyses

#### Total Se Determination

The total Se concentration in plant samples was measured using inductively coupled plasma mass spectrometry (ICP-MS; model iCapQ, Thermo Fisher Scientific, Bremen, Germany) following microwave-assisted acid digestion. Approximately 0.2 g of plant material was weighed into perfluoroalkoxy vessels and mixed with 6 ml of concentrated nitric acid (HNO_3_) before microwave heating (Model Multiwave 3000, fitted with a 48-place rotor; Anton Paar, Graz, Austria). The digested samples were then made to 20 ml final volume using Milli Q water and further diluted 10-fold with 2% HNO_3_ prior to analysis.

#### Speciation Analysis

An enzymatic hydrolysis method was employed to prepare the foliar-treated leaves and wheat grain samples for Se speciation analysis. The method of analysis was adapted from Muleya et al. (29). Four Se species were assayed: selenate, selenite, seleno-L-cysteine (SeCys), and seleno-L-methionine (SeMet). A multistandard solution (10 ml) containing the four Se species nominally at 5 μg L^−1^ concentration was prepared by diluting stock solutions of ^77^Se^IV^ and ^77^Se^VI^ (1,000 mg L^−1^) and SeCys and SeMet (100 mg L^−1^); the stock solutions with organic Se were prepared by dissolving the individual salts in Milli Q water. The Se concentrations of the individual Se species standards were verified by analysis (direct aspiration) using ICP-MS, with measured Se concentrations of 6.47, 5.37, 5.28, and 5.30 μg L^−1^, respectively.

Five millilitres of an enzyme solution containing 0.02 g protease K (Type XIV ≥ 3.5 units mg^−1^ solid from *Streptomyces griseus*) and 0.01 g lipase (Type VII ≥ 700 units mg^−1^ solid from *Candida rugosa*) was added to plant samples (0.2 g) in centrifuge tubes. The samples were incubated in the dark and shaken in a water bath set at 60 rpm at 37°C for 24 h; after incubation, they were centrifuged at 3,000 g for 30 min and filtered through 0.25 μm filters. Enzymatically-hydrolysed samples that were not immediately analysed were stored at 4°C in the dark. Selenium speciation analysis was undertaken using coupled HPLC-ICP-triple quadrupole-MS (ICP-QQQ-MS) instruments (Supplementary Table 1). The ICP-QQQ-MS was operated in oxygen cell mode to enable mass shifting of the Se isotopes and thereby minimise interferences; thus, ^77^Se was mass shifted to m/z 93 and ^80^Se to m/z 96. Standards were run after every block of 12 samples to monitor drift and enable correction of sample concentrations (22).

Sample processing was undertaken using a version of Chromeleon (Dionex) chromatography software operating within the iCapQ Qtegra software; the peaks generated by the individual Se species were manually integrated for peak area. Raw intensity data (integrated counts-per-second, iCPS) were then imported from the ICP-QQQ-MS at mass:charge (m/z) ratios of 93 and 96.

The enzymatically-hydrolysed plant samples were also analysed for a total ^80^Se and ^77^Se by ICP-MS, following a 1:10 dilution of the original enzyme extracts with 2% HNO_3_ acid. The final concentrations of the individual Se species were calculated from the proportion of the total extract Se that was measured as the peak area of the individual species, as described in Mathers et al. (22). For example, the concentration (μg L^−1^) of SeMet (at m/z 93 and 96) was calculated from Equation 1:


(1) $$\begin{array}{r} \text{SeMe}{\text{t}}_{\text{conc}}=\;\frac{\text{SeMe}{\text{t}}_{\text{cps}}\;}{\sum \text{specie}{\text{s}}_{\text{cps}}\;}\times \text{ S}{\text{e}}_{\text{tot},\text{enz}} \end{array}$$

where SeMet_cps_ is the peak intensity (iCPS) of SeMet and ∑species_cps_ the sum for all four species (SeMet_cps_, SeCys_cps_, Se${}_{\text{cps}}^{\text{IV}}$, and Se${}_{\text{cps}}^{\text{VI}}$), and Se_tot, enz_ is the total Se concentration (μg L^−1^) measured in the enzyme-hydrolysed extracts.

The concentration of individual Se species and total Se concentrations was then converted to a gravimetric basis using the dry weights of individual samples and the volume of the different extracts.

### Quality Control

Replicate samples of standard reference material (tomato leaves NIST 1573a) were acid digested and analysed for total Se by ICP-MS to provide quality assurance for the analysis of the plant samples. The recovery of Se in the reference material was within 100 ± 10% of the certified value (certified value 0.0543 mg kg^−1^; analysed value 0.0488 mg kg^−1^ Se)

The extraction efficiency of the enzyme (E_ext_) was calculated as follows (Equation 2).


(2) $$\begin{array}{r} {\text{E}}_{\text{ext}}\;=\;\frac{\text{S}{\text{e}}_{\text{tot},\text{enz}}}{\text{S}{\text{e}}_{\text{tot},\text{acid}}}\times \;100 \end{array}$$

where Se_tot, acid_ is the total Se concentration measured by acid hydrolysis for individual samples (μg L^−1^).

### Statistical Analyses

The effects of the different fertilisation treatments on grain yield and Se concentrations in plants were determined using the ANOVA procedure in SPSS (IBM SPSS Statistics for Windows, Version 24.0, IBM Corp, Armonk, New York), with a significance threshold of 5%. Duncan's and Tukey's *post-hoc* tests were used to compare treatment means.

## Results

### Biomass Yield

The yield of plants, calculated as the dry weight of the aboveground biomass, increased significantly with time but no significant differences in yield were observed among the different Se treatments (Table 2).

### Selenium Distribution in Plants

**Figure 1:**
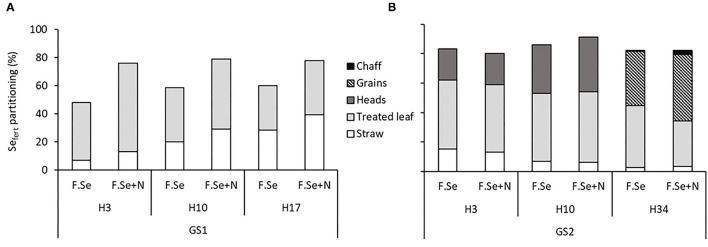
Partitioning of applied foliar Sefert (±N) in the aboveground biomass of plants as a function of harvest time [3, 10, and 17/34 days after application at stem elongation, GS1 **(A)**/heading stage, GS2 **(B)**]. The recovery of applied Sefert was calculated as the percentage of applied fertiliser Se that was recovered in the aboveground plant parts. Note that at H34, wheat heads were hand threshed to separate grains and chaff.

The recovery of Se_fert_ in plants harvested at GS1 was > 50%, even after 3 days following application (Figure 1A). The partitioning data showed that the majority (>63%) of the applied Se_fert_ was measured in the treated leaves up to 10 days after application, which decreased to <50% by day 17, suggesting mobilisation from the leaf to the straw. This mobilisation was more efficient for F.Se+N-treated plants, suggesting that the inclusion of N to foliar Se solutions improved the transfer of Se from the point of application to the rest of the aboveground biomass (Figure 1A).

For GS2 samples (122–153 DAS), the aboveground biomass was further separated into straw, heads, and leaves (Figure 1B). With high Se_fert_ recoveries in the aboveground biomass, limited losses of the applied foliar Se fertilisers to the environment were observed. This was confirmed by Se_fert_ levels in the foliar rinses being below analytical detection limits (data not shown for brevity). Within 3 days of application, 43 ± 0.98% Se_fert_ was translocated from the point of application to the rest of the aerial plant parts, which was equally distributed between the wheat heads and the straw. At the last sampling time (153 DAS), this translocation increased to 56 ± 5.2%, with heads, especially the grains, accumulating significantly more Se_fert_ than straw (*p* < 0.05). No significant differences in the recovery of Se_fert_ in the aboveground biomass of plants were observed between foliar Se (±N) treatments at GS2.

**Figure 2:**
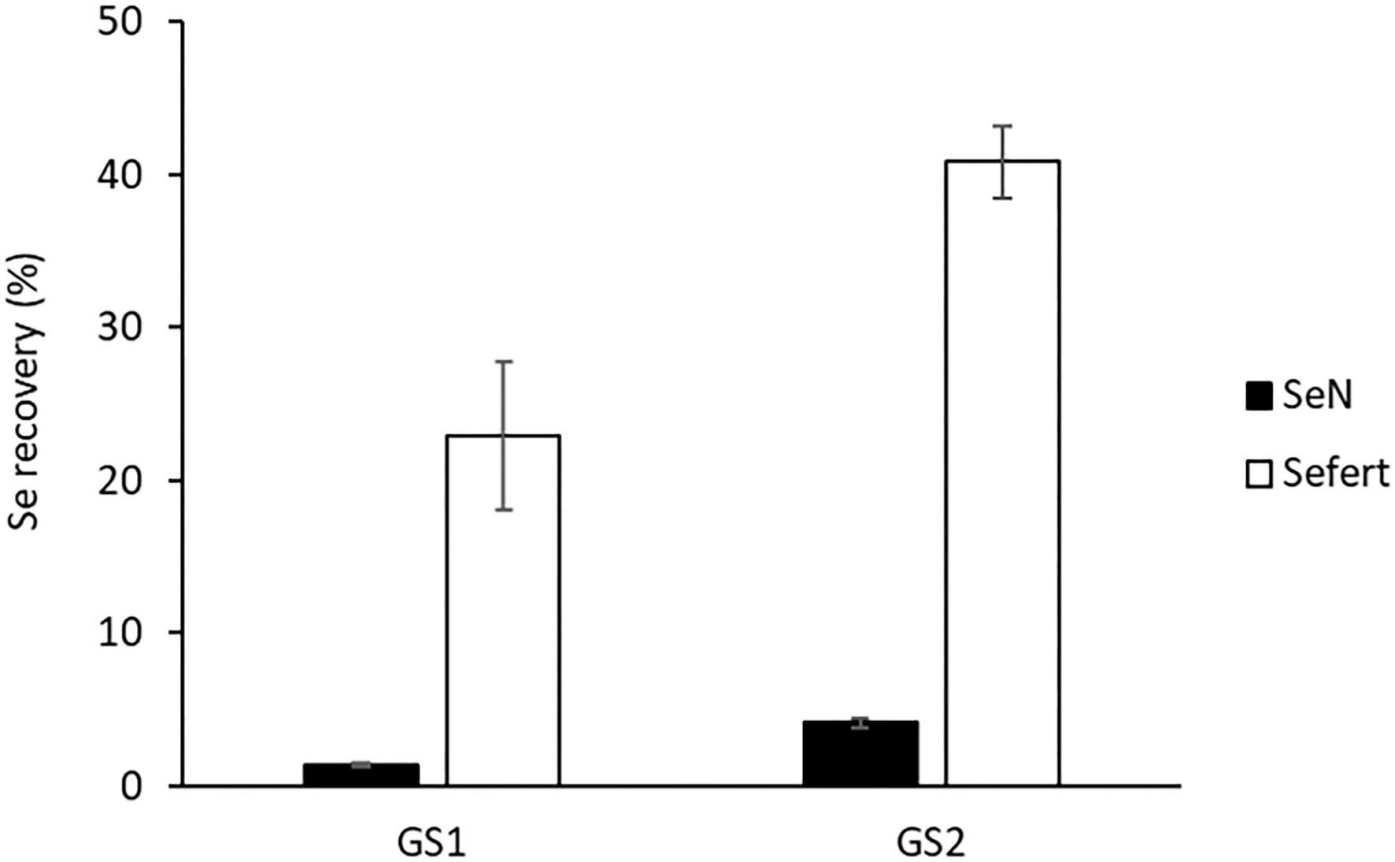
The recovery of native Se (Se_N_) and fertiliser-derived Se (Se_fert_) in the aboveground biomass of plants (excluding the foliar-treated leaves) harvested at stem elongation (GS1) and the heading stage (GS2). The recovery is the percentages of soil Se or fertiliser-applied Se that was recovered in the aboveground plant parts. Error bars represent SEs (*n* = 4).

In comparison to Se_fert_ recovery in plants, the recovery of native Se (Se_N_) in the aboveground plant biomass was much lower (Figure 2), indicating that the applied Se fertiliser was more available for plant uptake than native soil Se. Plants harvested at GS2 had accumulated significantly more Se_N_ (4.14 ± 0.33%) than those harvested at GS1 (1.37 ± 0.17%), likely because GS2 plants had a longer contact time with the native Se pool.

### Effect of N Addition in Foliar Se Solutions on Se_fert_ Uptake

The inclusion of N with foliar Se solutions led to greater Se uptake compared with foliar Se application on its own when applied at GS1, but this was not apparent at GS2 (Table 3). At GS2, the translocation of Se_fert_ into the wheat plants increased with growth time but was not affected by the addition of N to the foliar Se formulations.

**Table 3:** The influence of *N* inclusion with foliar Se solutions and harvest time on the accumulation of Se from the fertiliser (Se_fert_) in the aboveground biomass (foliar-treated leaves excluded) (SE in brackets; *n* = 4).

**Time after Se_**fert**_ application (*d*)**	**Se**_**fert**_ **uptake (μg pot**^**−1**^**)**			
	**GS1**		**GS2**	
	**–*N***	**+*N***	**–*N***	**+*N***
3	0.495 (0.03)	0.946 (0.36)	2.40 (0.40)	2.31 (0.30)
10	1.44 (0.28)	2.11 (0.49)	2.85 (0.11)	3.12 (0.32)
17/34^‡^	2.06 (0.25)	2.84 (0.55)	2.90 (0.21)	4.00 (0.51)
**Two-way ANOVA**
Day	<0.05		<0.05	
*N*	<0.10		ns	
Day*N	ns		ns	

“*ns” denotes non-significant interactions at p <0.05*.

‡ *The last sampling was done 17 and 34 days after Se_fert_ application at GS1 and GS2, respectively*.

**Figure 3:**
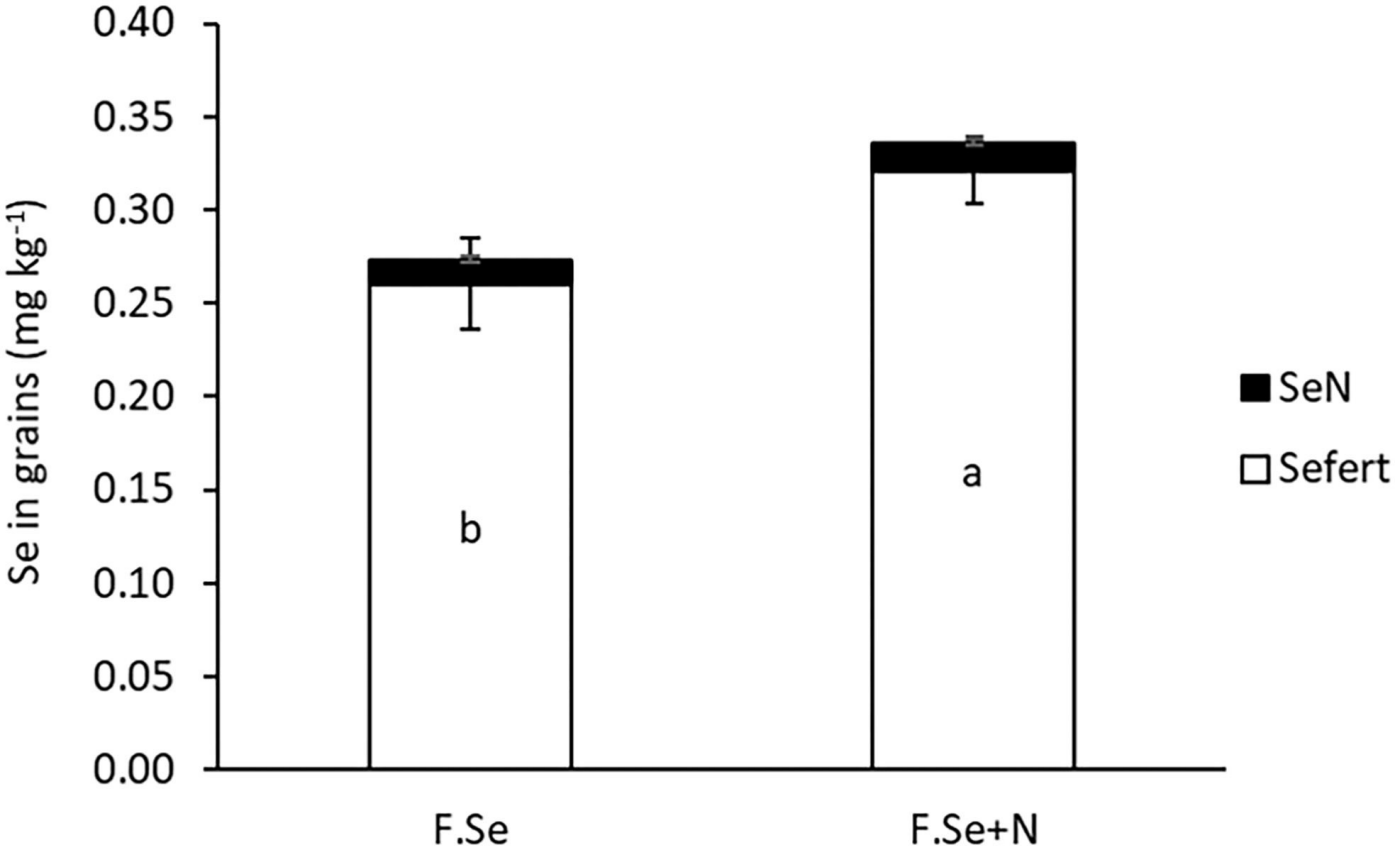
The concentration of native and fertiliser-derived Se in wheat grains. Error bars represent SEs (*n* = 4). “a” and “b” represent statistical differences in means at the *p* = 0.05 level.

The effectiveness of N inclusion in foliar Se fertilisers was observed in the grains (Figure 3). The average grain Se concentrations for foliar Se+N were 0.26 ± 0.02 and 0.32 ± 0.07 mg kg^−1^, which accounted for 44 and 54% of the applied Se transferred to the grain, respectively.

### Se Speciation in Grain

Protease hydrolysis extracted >60% of the total Se concentration in the wheat grain (Equation 2). Selenomethionine was the most abundant species in the wheat grain, accounting for >90% of the total Se_fert_ in the grain. A small amount of Se^VI^ (<10% of the total Se_fert_) was also detected in the grain, and no Se^IV^ or SeCys was measured, irrespective of Se treatments (Table 4). The inclusion of N in foliar Se solutions led to a significantly higher SeMet concentration in grains (0.21 mg kg^−1^), compared with F. Se-only fertilisation (0.16 mg kg^−1^) (Table 4).

**Table 4:** The distribution of the extracted Se species in wheat grain expressed as mean concentration or as % of total extracted grain Se (SE in brackets, *n* = 4).

**Treatments**	**Se species in grain**					
	**SeMet**		**Se** ^**VI**^		**Se^**IV**^**	**SeCys**
	**mg kg^**−1**^**	**% of total**	**mg kg^**−1**^**	**% of total**		
F.Se	0.16 (0.02)	92 (0.3)	0.014 (0.00)	8.3 (0.3)	n.d	n.d
F.Se+N	0.21 (0.02)	94 (2.3)	0.010 (0.00)	6.3 (2.3)		

“*n.d.” denotes non-detectable concentrations of species*.

#### Se Speciation in Leaves Treated With Foliar Se (±N)

**Figure 4:**
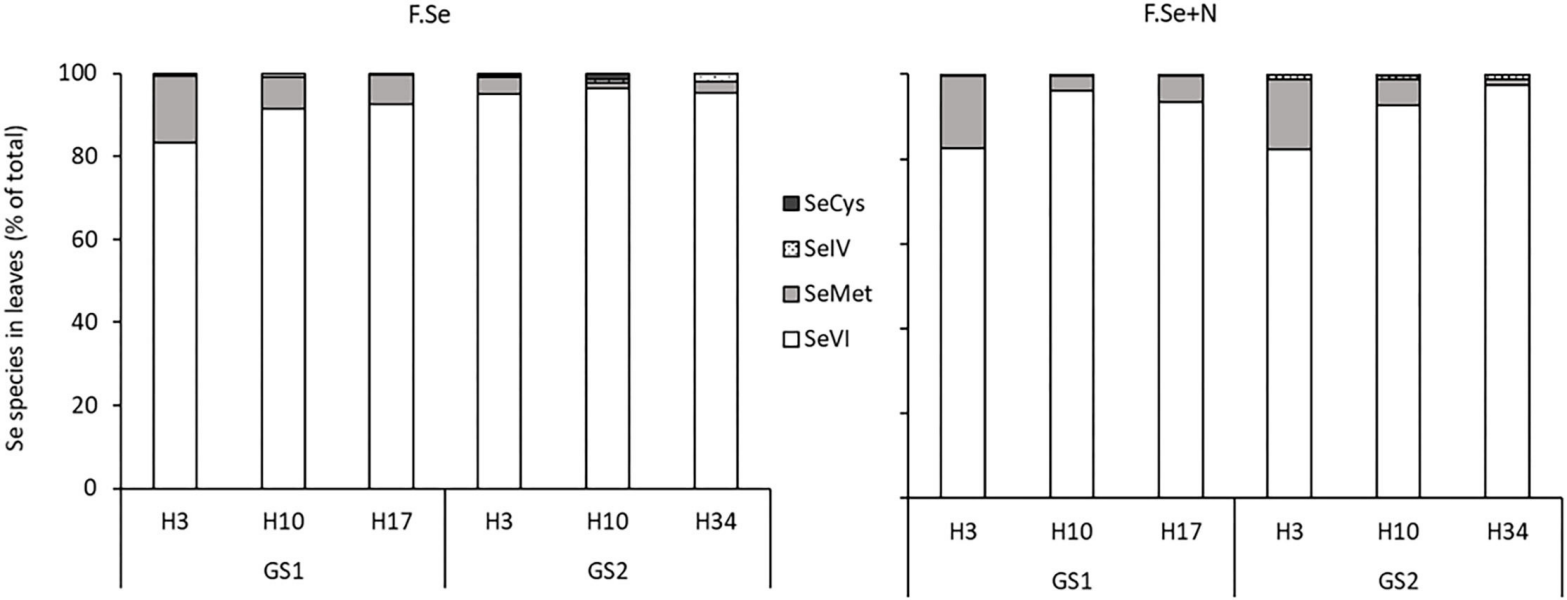
The distribution of Se species as a percentage of the total Se in leaves that were treated with F.Se and F.Se+N and harvested at different times following application at stem elongation (GS1) and heading (GS2).

The protease hydrolysis extracted, on average, 72 ± 2.4% of the total Se_fert_ in the foliar-treated leaves. The main species identified in the extracts were Se^VI^ (91 ± 2.0%) and SeMet (8.0 ± 1.9%); negligible concentrations of Se^IV^ and SeCys (<2% of the Se_fert_ in the leaves) were measured (Figure 4). For both F.Se and F.Se+N treatments, the distributions of the Se species in wheat were similar.

**Figure 5:**
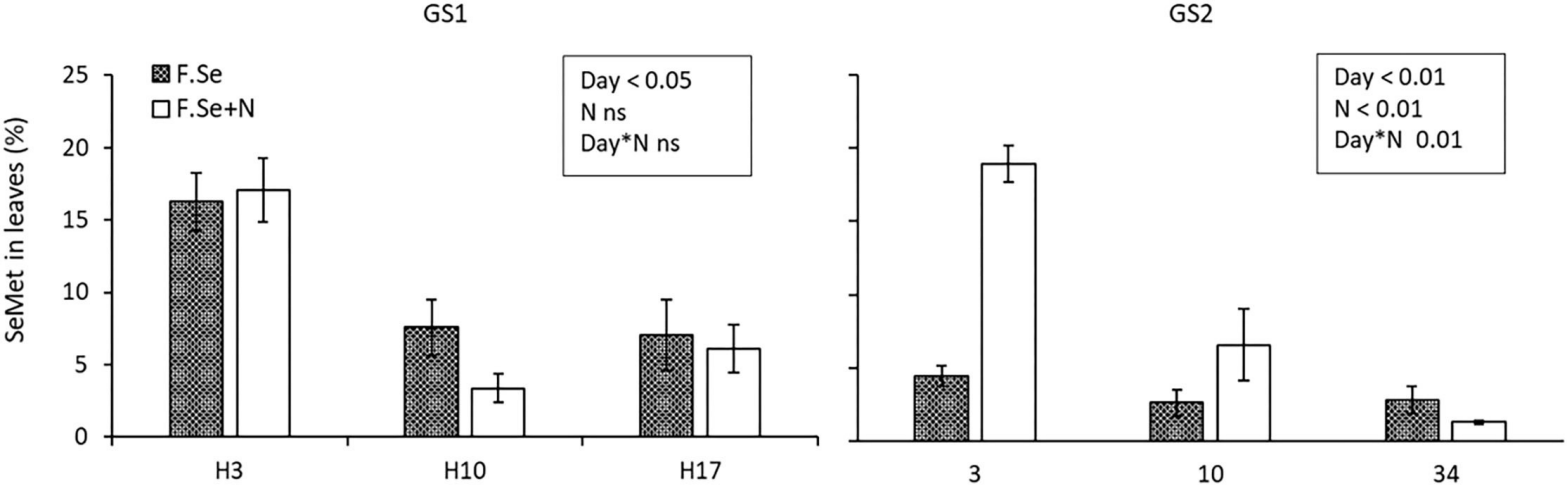
The concentration of SeMet in leaves that were treated with foliar Se and foliar Se+N at stem elongation (GS1) and heading stage (GS2). Results show averages and error bars represent SEs (*n* = 4). The *p*-values displayed on the graph show statistical significance from a two-way ANOVA.

Selenate was the most abundant species in the foliar-treated leaves, and its proportion did not change significantly over the 153-day experimental period (91 ± 1.6%) (Figure 4). By comparison, the proportion of SeMet decreased significantly with harvest time, in a similar way for both GS1 and GS2, which suggests more rapid mobilisation of SeMet to the rest of the plant compared with other Se species (Figure 4). The influence of N on the SeMet concentration in leaves and its translocation was observed only at GS2 (Figure 5). The application of F.Se+N led to significantly more transformation of the applied inorganic Se into SeMet, resulting in more rapid translocation of SeMet away from the application leaf (Figure 5).

## Discussion

Plant biomass was not influenced by the different Se treatments (Table 2), which was expected given that Se does not play an essential role in plant nutrition. Although Se can mitigate stress in plants by stimulating the activity of antioxidants (30), its essentiality in higher plants is not proven (31).

The Se concentrations of control plants and grains in the experiment were below the lower threshold of adequacy in the diet of 0.1 mg kg^−1^ dry matter (32), suggesting very low available Se levels in the soil used in this experiment. Accordingly, very low levels of Se_N_ (0.015 mg kg^−1^) were observed in the plants and the majority of the Se in the wheat originated from the fertiliser source (Se_fert_) (Figure 1). Similar findings were observed by Muleya et al. (29), who reported a Se_N_ concentration range of 0.01–0.03 mg kg^−1^ in three crops grown in Se-deficient soils in Malawi.

The application of Se by the foliar method appears to be very successful in promoting Se uptake by wheat, with minimal losses to the environment; recoveries of Se_fert_ in crops ranged from 60 to 100% (Figure 1). Such recoveries evidenced the higher effectiveness of foliar fertilisers compared to soil-applied ones for biofortification (16). Broadley et al. (33) and Mathers et al. (22) recovered <20% of applied Se in wheat grain, following 10 g ha^−1^ soil-application of Na_2_SO_4_ in the UK. Similarly, Lyons et al. (34) and Curtin et al. (35) recovered 13.5 and 17.0% in wheat grain, respectively, from the soil application of Se^VI^. The efficiency of foliar micronutrient fertilisers can reportedly be further improved by adding small amounts of N in the foliar solutions. Aciksoz et al. (36) observed that the addition of 1% N-urea (w/v) to foliar Fe fertilisers increased grain Fe concentrations, potentially by facilitating the cuticular penetration of foliar-applied Fe. Similarly, in a previous study, we observed that the addition of 2% w/v N in the form of urea or urea ammonium nitrate (UAN) to the foliar Se solution significantly improved grain Se concentrations compared to foliar Se application on its own (20). Effectively, a clear, positive effect of foliar Se coapplication with N on plant and grain Se concentration was observed (Table 3); the mechanisms for this positive effect appeared to differ according to the timing of application. At an early growth stage (GS1), the presence of N in foliar Se solutions significantly increased Se accumulation in the aboveground biomass of the plants (Table 3), potentially due to improved absorption of the applied Se_fert_ through the cuticle of the wheat leaves and/or improved assimilation of the applied inorganic Se into organic compounds and subsequent translocation. Since the speciation analysis of the foliar-treated leaves at GS1 showed no effect of N on the formation of SeMet (Table 4), it is likely that a physiological mechanism was responsible for the greater efficiency of the foliar Se+N fertilisers.

At a later growth stage (GS2), the fertiliser formulations were equally effective in raising plant Se concentrations, but those fertilised with F. Se+N had higher Se concentrations in the grain (Figure 3), suggesting improved translocation of Se from the point of application to the grain. Speciation analysis of the foliar-treated leaves suggested that N in the foliar Se solution improved the conversion of Se^VI^ to SeMet in the leaves, which was then more rapidly translocated to the grain (Table 4). Given that N and Se share a common metabolic pathway in plants (30), the coapplication of foliar Se with N at a stage where plants have a high metabolic activity (GS2) most likely affected the rate of Se assimilation and translocation within the plant. Hence, the coapplication of foliar Se with N at the heading stage was highly beneficial in improving the nutritional status of the plant, which has important implications for biofortification.

The target grain Se concentration range desired for biofortification, without running the risk of toxic effects, is >0.1 and <1 mg kg^−1^ (32). The application of foliar Se (±N) in this study increased grain Se concentrations to 0.25–0.3 mg kg^−1^ (Figure 3), which is optimal for biofortification, based on an RDI of 55–65 μg day^−1^ (17, 37). The application of foliar Se+N resulted in significantly higher Se concentrations in the grain compared with the foliar Se application on its own (Figure 3), of which >90% was in the highly bioavailable SeMet form (Table 4). These findings confirmed our previous results, whereby the application of F.Se+N doubled the concentration of Se in grain compared with F.Se only (20). It is worth noting that this experiment was carried out under controlled conditions, whereby plants were grown in a glasshouse and foliar fertilisers were applied in a precise manner. This could explain why the recovery of foliar-applied Se fertilisers in plants (>60% at GS1 and >96% at GS2) was considerably higher than those where foliar fertilisers were applied in outdoor conditions. For example, Ducsay et al. (38) recovered 13–15% of Se in grain following foliar application of 10 g ha^−1^ Se^VI^ to wheat in small field experiments. Nevertheless, this is, to the best of our knowledge, the first study to map the transformation and translocation of Se in wheat following its application with and without N at different growth stages and, hence, provide practical information about ways to optimise foliar Se fertilisations.

## Conclusions

Applying foliar Se, irrespective of the formulation, at 10 g ha^−1^ equivalent brought grain Se concentration to a level high enough to be considered adequate for biofortification. Whether applied at an early or a late growth stage, foliar Se fertilisers can be made more efficient by coapplication with 2% w/v N as urea. The application of foliar Se with N to young wheat plants improved its absorption through the leaves, thereby reducing the window of opportunity for fertiliser Se to be lost to the environment either by volatilisation or by leaf runoff. At a later growth stage, the inclusion of N in foliar Se solutions improved the transformation of applied inorganic Se into bioavailable SeMet, which was then more rapidly translocated from the point of application to the grain. From the current study, it appears that the coapplication of foliar Se with 2% N-urea at the heading stage significantly increased the concentration of bioavailable Se in the grain. Farmers could use such information to optimise fertilisation strategies and minimise losses to the environment.

## Data Availability Statement

The raw data supporting the conclusions of this article will be made available by the authors, without undue reservation.

## Author Contributions

CR, SY, and EB: conceptualisation, data analysis and interpretation, and writing the review and editing. CR: experimental setup, data collection, analysis and interpretation, and writing. MM and FD: data analysis and interpretation and writing the review and editing. All authors contributed to the article and approved the submitted version.

## Conflict of Interest

The authors declare that the research was conducted in the absence of any commercial or financial relationships that could be construed as a potential conflict of interest.

## Publisher's Note

All claims expressed in this article are solely those of the authors and do not necessarily represent those of their affiliated organizations, or those of the publisher, the editors and the reviewers. Any product that may be evaluated in this article, or claim that may be made by its manufacturer, is not guaranteed or endorsed by the publisher.
