# Supplementary figures and images for: Using 77Se-Labelled Foliar Fertilisers to Determine How Se Transfers Within Wheat Over Time
Source: Front Nutr. 2021 Oct 15;8:732409. doi: 10.3389/fnut.2021.732409 (PMC8554058; doi:10.3389/fnut.2021.732409)

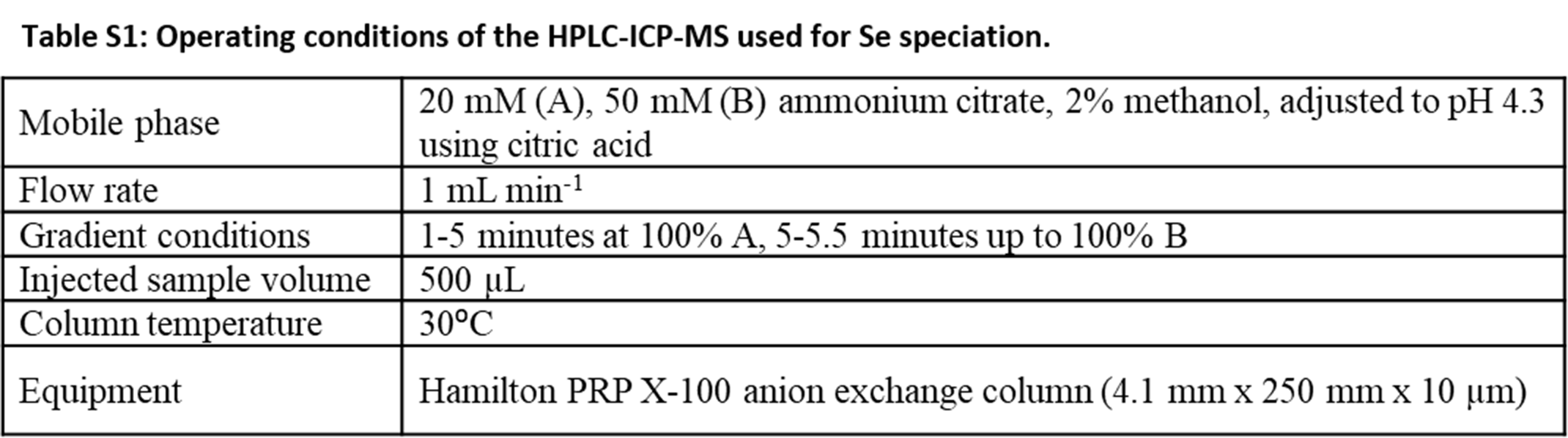

Supplement: Supplementary file 1 [file Image_1.TIF]
